# Cefsulodin and Vancomycin: A Supplement for Chromogenic Coliform Agar for Detection of *Escherichia coli* and Coliform Bacteria from Different Water Sources

**DOI:** 10.3390/microorganisms10122499

**Published:** 2022-12-16

**Authors:** Michael Schalli, Sarah Maria Inwinkl, Sabine Platzer, Rita Baumert, Franz F. Reinthaler, Petra Ofner-Kopeinig, Doris Haas

**Affiliations:** 1 Department for Water-Hygiene and Micro-Ecology, D&R Institute of Hygiene, Microbiology and Environmental Medicine, Medical University of Graz, 8010 Graz, Austria; 2 Institute for Medical Informatics, Statistics and Documentation, Medical University of Graz, 8010 Graz, Austria; 3 Applied Hygiene and Aerobiology, D&R Institute of Hygiene, Microbiology and Environmental Medicine, Medical University of Graz, 8010 Graz, Austria

**Keywords:** *Escherichia coli*, coliform bacteria, drinking water, bathing water, mineral water, Vancomycin, Cefsulodin

## Abstract

Background microorganism growth on Chromogenic Coliform Agar (CCA) can be challenging. For this reason, an alternative method with a Cefsulodin/Vancomycin (CV)-supplemented CCA should be evaluated regarding its application in water from different sources. CCA supplemented with CV was validated according to ÖNORM EN ISO 16140-4:2021 using water from natural sources in Styria, Austria. Results show that the alternative method using the supplemented CCA has similar values in relation to sensitivity (82.2%), specificity (98.6%) and higher selectivity (59%) compared to the reference method. Repeatability and reproducibility were acceptable for the alternative method and showed similar results with the reference method. The alternative method shows a very low false positive rate and a low false negative rate paired with good performance regarding the inclusion study. The exclusion study shows the advantage of our method by suppressing background microorganisms and facilitating the process of enumeration of *Escherichia coli* and other coliform bacteria on CCA plates. *Aeromonas hydrophila* and *Pseudomonas aeruginosa* growth was inhibited using the supplement. To conclude, the coliform CV selective supplement combined with CCA is an appropriate tool for coliform bacteria detection in water samples.

## 1. Introduction

**Figure 1:**
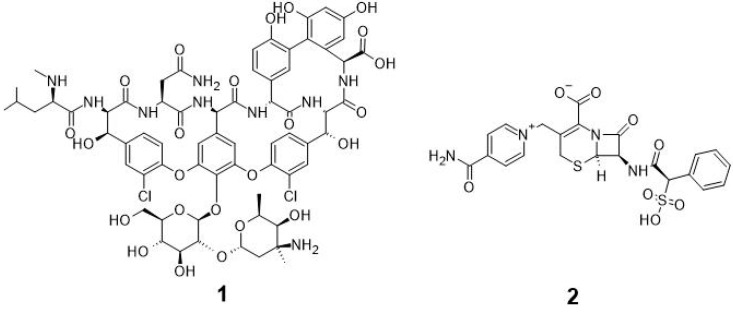
Chemical structures of compound **1** (Vancomycin) and compound **2** (Cefsulodin).

Drinking water contamination is a problem which can cause waterborne outbreaks and severe damage to the health of its consumers [1,2,3]. Bacterial contamination of groundwater, wells and surface water caused by faecal sources can be found worldwide in urban regions and regions with intensive agriculture [4,5,6]. Water supplies in poorer countries more frequently show bacterial contamination with thermotolerant coliform bacteria and *Escherichia coli* (*E. coli*) [7,8]. The analysis of water for consumption as well as for use in public baths is of great interest for consumers as well as for water suppliers. In order to determine the presence of faecal contamination in water, *E. coli* is used as an indicator. Coliform bacteria can reveal problems regarding the water transport system [9,10,11]. Different approaches for the detection of *E. coli* in water resources ranging from multi-tube fermentation to biosensor-based solutions have been developed in the past [12,13,14,15]. In their validation study, Lange et al. [16] describe the usage of Chromogenic Coliform Agar (CCA) for the enumeration of coliform bacteria and *E. coli*. The study describes a well-developed method for distinguishing between *β*-D-galactoside processing coliform bacteria and *E. coli*, which is in addition capable of cleaving *β*-D-glucuronides. The authors indicate that this method is not suitable for surface water and shallow well waters because of bacterial background growth. Accompanying microorganism growth in water samples can lead to overloaded uncountable CCA plates. In order to circumvent this problem, CCA plates have to be modified to suppress background microorganisms and make enumeration of target bacteria easier. In 2009, the Spanish government approved a method for the detection of *E. coli* and coliform bacteria based on an internal study, developed by Ribas et al. [17,18]. In the past, different antibiotic-resistant bacteria were isolated from natural sources [19,20,21,22]. Multidrug resistance is a major problem in medicine and occurs because of intensive usage of antibiotics in the last decades [23,24]. For better detectability, Vancomycin and Cefsulodin can act as suppressors for accompanying microorganism growth, giving *E. coli* and other coliform bacteria an advantage to grow on the selected media [25]. Cefsulodin/Vancomycin (CV)-assisted modified Tryptone Soy Broth was used to reduce accompanying microorganisms for detection of verotoxine producing *E. coli* (VTEC). Growth of different non-VTEC bacteria was tested with different combinations and concentrations of antibiotics. CV-supplemented media was found to give the most promising results for reduction of non-target bacteria [26]. The current method for the detection and enumeration of *E. coli* and coliform bacteria in water analysis using CCA is shown in ÖNORM EN ISO 9308-1:2017 [27]. The validation of our alternative method provides a possibility to optimize the existing method by an appropriate adaptation for comparable results in which validation parameters according to ÖNORM EN ISO 16140-4:2021 [28] are used. ÖNORM EN ISO 9308-1:2017 [27] describes a procedure for sample preparation where volumes of 10 mL to 100 mL of water are filtered through a membrane with subsequent cultivation on CCA at 36 ± 2 °C for 21 to 24 h. Specific bacterial enzymes lead to pinkish/red colonies indicating coliform bacteria and blueish/purple colonies indicating the presence of *E. coli*. The red colouring is due to bacterial *β*-D-galactosidase, and the additional presence of *β*-D-glucuronidase activity colours colonies blue. The presence of accompanying microorganisms can inhibit growth of the target bacteria as well as give false positive results, which makes it more difficult for selective colony counting. *β*-D-galactoside processing enzymes, present in *Aeromonas* spp. ubiquitous to water [29], also lead to red colonies on CCA. Bioactive small molecules, which are part of the huge class of carbohydrate derivatives, play an important role in medicine [30,31,32]. To inhibit the growth of Gram-positive bacteria, Vancomycin **1**, a carbohydrate derivative, which interrupts cell wall formation and crosslinking of parts of the cell wall, shown in Figure 1, can be used as a supplement agent [33]. Adding cephalosporins such as Cefsulodin to the media will inhibit the growth of *Pseudomonas aeruginosa*, *Aeromonas* spp. and other oxidase-positive organisms [34]. The chemical structure of Cefsulodin **2**, shown in Figure 1, enables fast penetration of the bacterial cell wall of Gram-negative bacteria and shows good stability against *β*-lactamases. *Enterobacteriaceae* such as the group of coliform bacteria are less affected by treatment with small concentrations of Cefsulodin [35].

The detection and identification of bacteria in water samples of different origins can be challenging. In this study we show that the detection and enumeration of *E. coli* and coliform bacteria by using membrane filtration and subsequent cultivation on CCA according to ÖNORM EN ISO 9308-1:2017 has limitations regarding the presence of a strong accompanying flora. Our approach shows the advantage of suppressing most of the background bacterial growth to make enumeration of colored bacteria colonies easier. Addition of a CV supplement to CCA should enhance the resolution for detection of *E. coli* and coliform bacteria.

## 2. Materials and Methods

### 2.1. General Methods for Sample Preparation

Preparation and membrane filtration, inoculation and incubation as well as counting of colonies were carried out according to ISO 8199:2018(E) [36].

Water samples (10–100 mL) were filtrated trough a mixed cellulose ester filter (47 mm diameter, 0.45 µm pore size, EZ-Pak, Merck Chemicals and Life Science GmbH, Vienna, Austria, EZHAWG474) under vacuum, and the filter was subsequently placed on a freshly prepared CCA plate for the application of the reference method, and the CV-supplemented CCA plate was prepared for the alternative method. After incubation for 24 h at 37 °C, counting of coloured *β*-D-galactosidase positive colonies (pink/red for coliform bacteria) and *β*-D-galactosidase-*β*-D-glucuronidase positive colonies (purple/blue for *E. coli*) was performed. 

### 2.2. Relative Trueness 

According to ÖNORM EN ISO 16140-4:2021 [28] for a quantitative factorial process, three different categories of water, including drinking water, water for public baths and highly mineralized water, with four characteristic types and origins, were selected. Within each category, twelve units were created and spiked with reference strains of *E. coli* and *Klebsiella aerogenes* (*K. aerogenes*) at three different levels of contamination. For each category with twelve units, we chose a low, middle and high bacterial contamination. The factorial investigation, according to the standard procedure, prescribes eight settings, whereby in each setting, four different factors were chosen (factor 1: technician; factor 2: culture media; factor 3: incubation temperature; factor 4: incubation time). Two batches of culture medium for the reference and alternative replicate at 34.5 °C or 37 °C for 21 or 24 h, respectively, were prepared. For each technician, 48 samples for each category were measured as described in Table 1.

### 2.3. Preparation of E. coli and K. aerogenes Contaminated Samples

From commercially available strains (Leibniz Institute, DSMZ-German Collection of Microorganisms and Cell Cultures GmbH, Braunschweig, Germany; American Type Culture Collection (ATCC), Manassas, VA, USA), material was freeze-dried in VIABANK^®^ vials containing 20 ceramic beads covered in cryopreservative solution and used for the preparation of contaminated samples [37]. One bead of the *E. coli* DSM 1103 reference strain contains approximately 10^6^ colony-forming units (CFU) whereas one bead of the *K. aerogenes* DSM 30053 reference strain contains 10^7^ CFUs. Each bead is diluted in 1 L of distilled water. After ten min, 1 mL of suspension is added to 9 mL of sample for subsequent membrane filtration. Due to the poor stability of the samples, prepared suspensions were filtrated directly after preparation. For each contamination level, the following amounts of bacteria-contaminated suspension were added to 10 mL of Ringer solution for filtration. For *E. coli* (200 CFU/mL to 300 CFU/mL): low level (70 µL), middle level (150 µL), high level (250 µL) and *K. aerogenes*: (800 CFU/mL to 900 CFU/mL): low level (25 µL), middle level (55 µL), high level (80 µL). After incubation for 21 or 24 h, CFU were counted. 

Values for pH and conductivity were measured according to the respective standards (ÖNORM EN ISO 10523:2012 [38] and ÖNORM EN 27888:1993 [39]) with a 712 Conductometer (Methrom^®^ Inula GmbH, Vienna, Austria) and a Memo-Titrator (Metrohm^®^ Inula GmbH, Vienna, Austria); the values can be found in Table 2. For the determination of the background microorganisms, 0.5 mL of undiluted water was plated on Columbia Agar with 5% Sheep Blood with a Drigalski spatula and incubated at 37 °C for 24 h. The artificial contamination was carried out using the *E. coli* DSM 1103 strain, which shows blue/purple colonies and *K. aerogenes* DSM 30053, which shows pink colonies on CCA.

### 2.4. Inclusion/Exclusion Study

The determination of inclusion and exclusion was performed according to ÖNORM EN ISO 16140-2:2016 [40]. Eighteen target organisms and twelve non-target organisms were taken from the D&R Institute of Hygiene, Microbiology and Environmental Medicine collection of microorganisms for proof of concept. Frozen samples (−80 °C) of the reference strains were cultivated on Columbia Agar with 5% Sheep Blood and incubated for 24 h at 37 °C. Each cultivated strain was inoculated into 1 mL of Peptone salt solution (bioMerieux^®^ Austria GmbH, Vienna, Austria), vortexed and adjusted to 1.5 × 10^8^ CFU/mL with a DensiCheck Plus (bioMerieux^®^ Austria GmbH, Vienna, Austria). For further membrane filtration a dilution series was performed, adding 20 µL of suspension to 980 µL of Peptone salt solution until 12 × 10^2^ CFU/mL was achieved. For each reference strain, 55 µL were transferred into 10 mL of Ringer solution and subsequently filtrated through a 47 mm diameter, 0.45 µm pore size, EZ-Pak, EZHAWG474 filter (Merck^®^ Chemicals and Life Science GmbH, Vienna, Austria,). After incubation for 24 h at 37 °C, CFUs were counted. Membrane filtration was performed for each reference strain in a dual approach with the reference and alternative methods. With respect to ÖNORM EN ISO 16140-2:2016 [40], an additional nonselective media for target organisms is required; Tryptic soy agar (VWR^®^ International GmbH, Vienna, Austria) was chosen for cultivation and comparison (results are shown in Table 4).

### 2.5. Sensitivity and Specificity 

Characterisation and comparison of the alternative method were carried out with routine samples according to DIN EN ISO 13843:2018 [41], investigating more than 20 different water samples from natural sources in a parallel study. After incubation on CCA and CCA including a CV supplement, colonies were counted and morphologically described by colour. After that, colonies were inoculated on Tergitol 7-lactose TTC agar (Oxoid^®^ Deutschland GmbH, Wesel, Germany) for determination of lactose fermentation. Inoculation on non-selective media Columbia Agar with 5% Sheep Blood (BD^®^ Austria GmbH, Schwechat, Austria) was carried out for further MALDI-TOF MS analysis. If no classification of the target colony was possible, Gram-staining was performed to obtain more information about the selected bacteria. Cytochrome oxidase testing was performed according to ÖNORM EN ISO 9308-1:2017 [27] for all pink and red colonies.

### 2.6. Preparation of CCA without Supplement

For the preparation of CCA without supplement, 88.4 g of Chromogenic Coliform Agar was added to 3 L of distilled water, heated and stirred in a Systec MediaPrep 20 Autoclave at 121 °C for 15 min. After cooling the material to 52 °C, 18 mL of diluted CCA was plated using the Integra MediaJet plating system. CCA plates were freshly prepared before usage.

### 2.7. Preparation of CCA with CV Supplement

The preparation of CCA with CV supplement was carried out using 88.4 g of Chromogenic Coliform Agar, which was added to 3 L of distilled water, heated and stirred in a Systec MediaPrep 20 Autoclave at 121 °C for 15 min. After cooling to 52 °C, six ampules of CV supplement (VWR^®^ International GmbH, Vienna, Austria, 928390NL) were diluted in 36 mL of distilled water and added to the agar. 18 mL of diluted agar with supplement was plated using the Integra MediaJet plating system for further sample preparation. CCA plates with CV supplement were freshly prepared before the membrane filtration step.

### 2.8. Bacterial Strains 

The following bacterial strains were used: *Enterobacter cloacae* DSM 30054, *E. coli* DSM 1103, *E. coli* DSM 1576, *E. coli* DSM 15210, *E. coli* DSM 5695, *E. coli* DSM 5923, *E. coli* DSM 10814, *E. coli* DSM 11250, *E. coli* DSM 18039, *E. coli* DH5 alpha, *E. coli* K12, *K. aerogenes* DSM 30053, *Klebsiella* sp. DSM 4798, *K. pneumoniae* ATCC 4352, *K. pneumoniae* DSM 789, *K. pneumoniae* DSM 26371, *K. pneumoniae* DSM 30104, *K. variicola* strain: ATCC 31488, *Aeromonas caviae* DSM 7323, *Aeromonas hydrophila* DSM 30187, *Acinetobacter baumannii* DSM 30007, *Acinetobacter calcoaceticus* DSM 30006, *Acinetobacter Iwoffii* DSM 2403, *Proteus mirabilis* DSM 788, *Pseudomonas aeruginosa* DSM 939, *P. aeruginosa* DSM 1117, *P. aeruginosa* DSM 1128, *P. aeruginosa* DSM 50071, *Salmonella enterica* subsp. *enterica* serotype Enteritidis DSM 17420, *Salmonella enterica* subsp. *enterica* serotype Typhi DSM 19587; Bacterial samples obtained from commercial resources (28 bacterial samples were obtained from Leibniz Institute, DSMZ-German Collection of Microorganisms and Cell Cultures GmbH, Braunschweig, Germany; two bacterial samples were obtained from American Type Culture Collection (ATCC), Manassas, Virginia, USA and transported in glass vials.

### 2.9. Culture Media

Chromogenic coliform agar (CCA), Tryptic soy agar (TSA) and coliform CV selective supplement were obtained from VWR^®^ International GmbH, Vienna, Austria. All culture media were prepared according to the manufacturer’s instructions. Tergitol 7 lactose TTC agar (OXOIPO5164A) was obtained by OXOID^®^ Deutschland GmbH, Wesel, Germany. Columbia Agar with 5% Sheep Blood (254071) was obtained from BD^®^ Austria GmbH, Schwechat, Austria.

### 2.10. Oxidase Test

Oxidase activity testing set (BD BBL^tm^ DrySlide Oxidase 231746) was obtained from BD^®^ Austria GmbH, Schwechat, Austria and used according to the manufacturer’s instructions.

### 2.11. Gram Staining

Gram staining was performed using IUL polystainer (10005300/768) and Gram-colours from Sigma-Aldrich^®^, Darmstadt, Germany (Grams crystal violet solution 1.09218.2500; Lugol’s solution 1.00567.2500 and Gram’s safranine solution 1.09217.2500). Microscopy was performed using a Carl Zeiss AG, Oberkochen, Germany, Axio-imager A1, according to the instructions of the manufacturer.

### 2.12. Data Analysis 

Calculation of sensitivity, specificity, selectivity, false positive rate and false negative rate was performed using Microsoft Excel. Accuracy profile studies, relative trueness studies, and inhouse precision were analyzed using the software MiBiVal from QuoData GmbH, Dresden, Germany. The analysis of variances was made for all contamination levels (for *E. coli* and *K. aerogenes*) between the reference method and the alternative method divided into low, middle and high contamination levels, as well as all contamination levels in between, and were found to be homogenous. The homogeneity analysis of data was performed using SAS V9.4. To assess equality of variances, Levene’s Test was used. All *p*-values ≤ 0.05 are considered as statistically significant; therefore, a *p*-value > 0.05 indicates equal variances, or homogenous data. 

### 2.13. MALDI-TOF VITEK^®^ MS 

Characterisation of target bacteria was performed using a Vitek system (bioMérieux^®^, Vitek^®^ MS 60313/09) according to the instructions of the manufacturer.

### 2.14. Water Samples

All water samples, derived from different water sources, (category α: one sample was public tap water, three samples were well water; category β: four samples were from public baths; and category γ: four types of mineral water) used in this study were obtained from public and private water supplies and baths in Styria (Austria). Water samples (n = 21) used for the determination of specificity and sensitivity were collected from public water supplies (tap water) in Styria.

## 3. Results and Discussion

### 3.1. Identification of Target Bacteria

**Figure 2:**
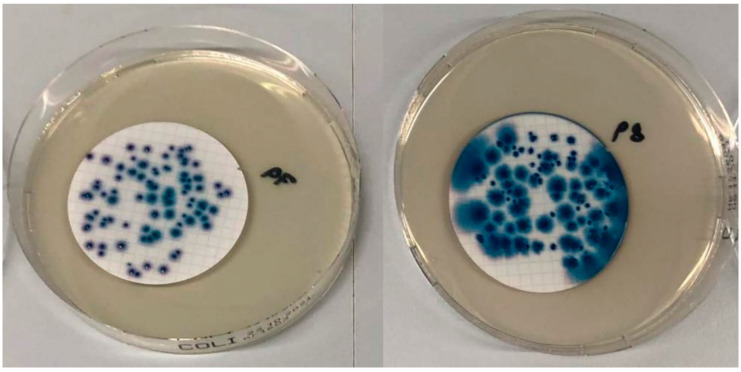
(**left**): *E. coli* on CCA with CV supplement: (**right**): *E. coli* on CCA without supplement.

**Figure 3:**
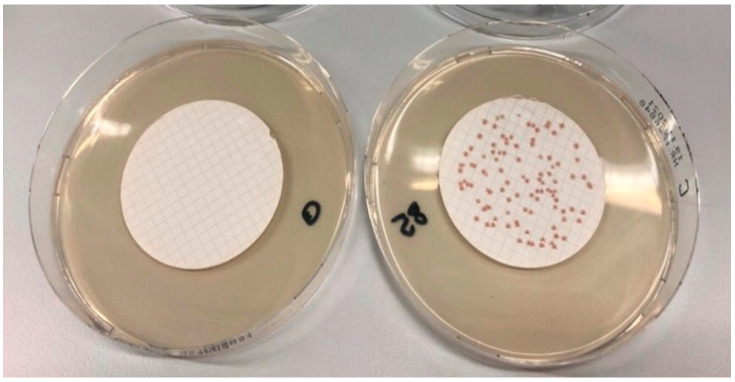
(**left**): *Aeromonas hydrophila* on CCA with CV supplement: (**right**): *Aeromonas hydrophila* on CCA without supplement.

Detection of *E. coli* and coliform bacteria using supplemented CCA containing Vancomycin and Cefsulodin as supplement was compared to the established procedure, introduced by Lange et al. in 2013 [16]. Validation of the new alternative method was carried out according to ÖNORM EN ISO 16140-2 (2016) [40]. Identification of target bacteria with alternative and reference methods led to blue colonies shown in Figure 2 for *E. coli* and red/pink colonies for coliform bacteria. Colonies grown on CCA without supplement (reference method) were dark blue and had a flat profile, whereas colonies on CCA with a Cefsulodin/Vancomycin supplement (alternative method) showed a purple/blue colouring and a defined curvature. Colony counting with higher contamination levels was easier with CV-supplemented CCA. Non-target bacteria (*Aeromonas hydrophila*) did not grow on CV-supplemented CCA, in contrast to the reference method where red colonies were found (shown in Figure 3).

### 3.2. Selectivity Studies

Performance testing of the two methods included the investigation of 21 water samples of different origin with different contamination levels. For each colony, morphology, cytochrome-oxidase-testing, lactose fermentation and MALDI-TOF VITEK^®^ MS (bioMerieux^®^ Austria GmbH, Vienna, Austria) analysis was performed. With this data, it was possible to associate each colony with one of four groups: (a) true positive, (b) false negative, (c) false positive and (d) true negative, as can be seen in supporting information in Table S1. The amount of true positive colonies was 148 out of 251 for the supplemented CCA, and only one colony was noticed to be false positive. Sensitivity is slightly lower in the alternative method, whereas the reference method shows a slightly lower specificity. Results for the alternative method show a sensitivity of 82.2% and specificity of 98.6%, which is a level similar to the values calculated for the reference method (sensitivity 85.3% and specificity 97.3%). Values for selectivity show a difference between both methods, with 37.9% for CCA without a supplement and 59.0% for CV-supplemented CCA. The false positive counting was very low, at 0.7% for the alternative method, but the false negative rate was 31.4% during the investigations. Calculated data, comparing both methods, are shown in Table 3. The present validation indicates that the alternative method is sensitive, selective and specific for counting *E. coli* and coliform bacteria derived from natural water samples. The summary of all detected and identified bacteria as well as the calculation of values is shown in the supporting information in Table S1.

### 3.3. Relative Trueness

**Figure 4:**
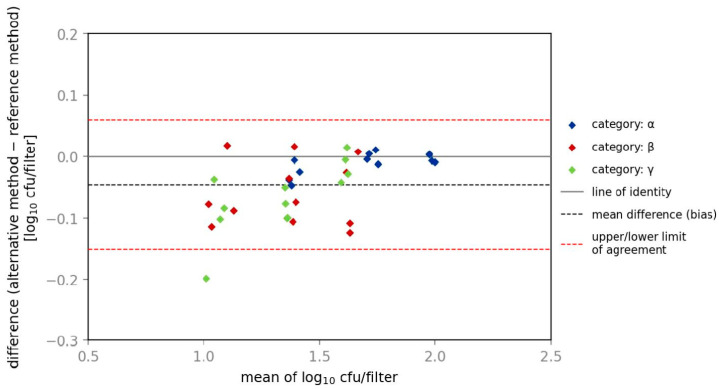
Bland–Altman plot of spiked samples with *E. coli*.

**Figure 5:**
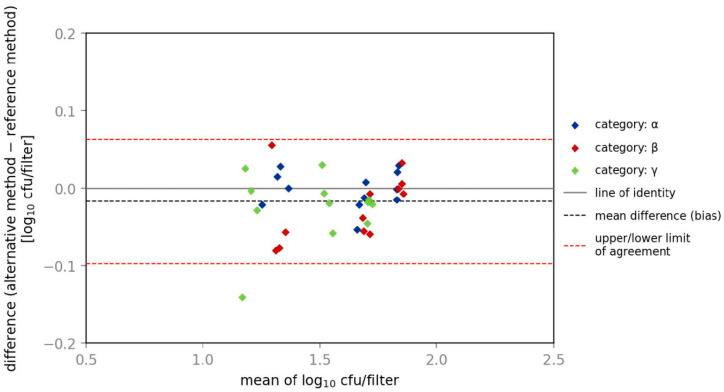
Bland–Altman plot of spiked samples with *K. aerogenes*.

Results for relative trueness show that similar ratios were obtained with the reference and alternative methods for investigation of *E. coli*, in all three categories (α, β and γ) at all three contamination levels. In Figure 4 and Figure 5, the Bland–Altman Plot provides information about the bias for categories α, β and γ and gives a graphical comparison of two different assays including identification of systematic differences between them. In categories β and γ the alternative method shows slightly lower amounts of CFU than the reference method with a ratio of 0.9.

**Figure 6:**
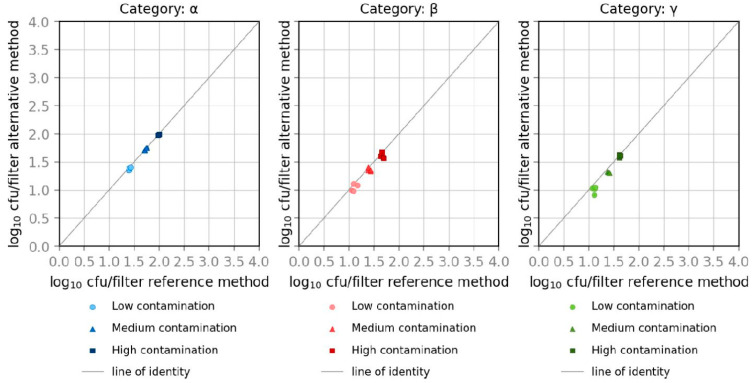
Youden plots of spiked samples with *E. coli*.

**Figure 7:**
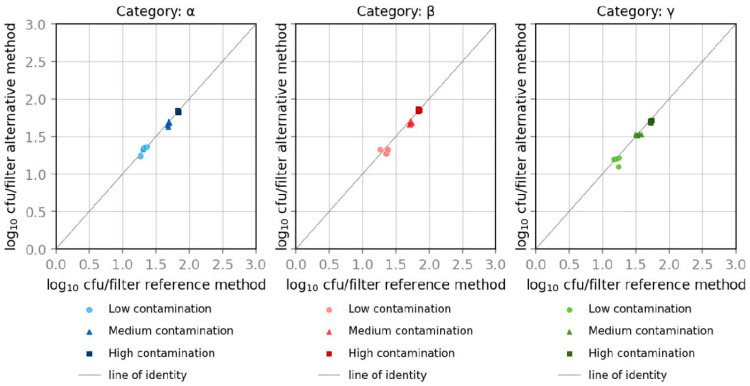
Youden plots of spiked samples with *K. aerogenes*.

The Youden Plot, which illustrates CFU in logarithmic steps, shows that results for all three contamination levels with *E. coli* and *K. aerogenes* are located in the range of the main diagonal and are similar to the reference method, shown in Figure 6 and Figure 7. Calculated data for *E. coli* and *K. aerogenes* show similar results according to the relative trueness of the alternative method. Data were calculated using the software Mibival (Quodata GmbH, Dresden, Germany). All measured values are around the mean and within the upper and lower acceptability limits for both measurements at all three levels of contamination.

### 3.4. Accuracy Profile Study

**Figure 8:**
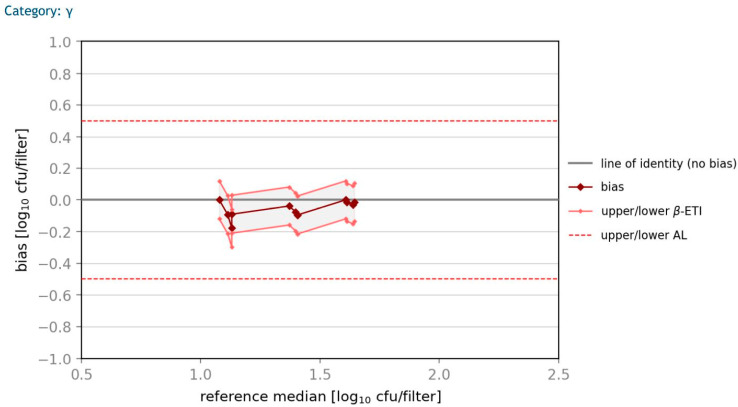
Accuracy profile of high-contamination sample with *E. coli*.

**Figure 9:**
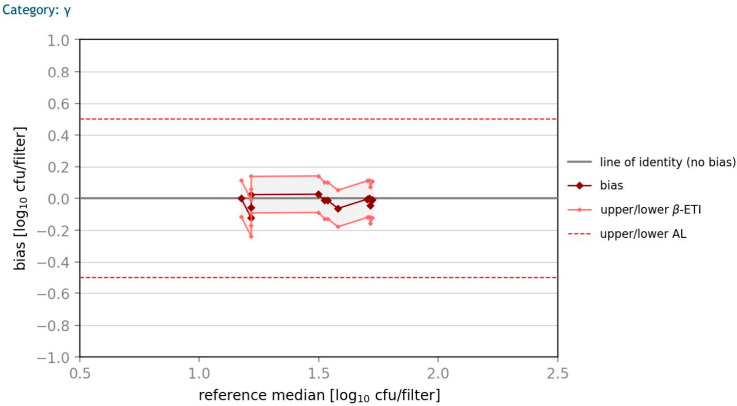
Accuracy profile of high-contamination sample with *K. aerogenes*.

According to ÖNORM EN ISO 16140-2 (2016) [40], an accuracy profile study should be done to show if the differences between the results of the two methods lie below the specified acceptability limits. All calculations are based on log_10_ transformed values, whereby the bias between the methods is plotted against the median of the reference method with the corresponding 80% prediction interval (*β*-ETI). In Figure 8 and Figure 9, plots for *E. coli* and *K. aerogenes*-contaminated samples with a high contamination level are shown. The *β*-ETI intervals of the spiked samples are within the acceptability limits for all six samples with an acceptability limit specified as AL = 0.5 log_10_ CFU/filter. An accuracy profile study showed similar results in all three categories for *E. coli* and *K. aerogenes*. The difference of reference value and the average result of log_10_ CFU/filter is very low for all calculations.

### 3.5. In-House Precision and Inclusion/Exclusion Study

In-house precision describes the distribution of measured single values around the median, which characterizes the precision of alternative methods. Standard deviations are important performance characteristics for in-house repeatability and in-house reproducibility, as for any measurement method. Reproducibility and repeatability were calculated for both methods with a repeatability for highly contaminated samples (*E. coli*) with 0.083 and 0.078 for *K. aerogenes* and similar results for reproducibility (high contamination with *E. coli* 0.083 and *K. aerogenes* 0.080). The alternative method showed a high precision for *E. coli* as well as *K. aerogenes*, comparable to the reference method. Results for the in-house precision calculations are shown in Table 3.

According to ÖNORM EN ISO 16140-4:2021 [28], determination of inclusion and exclusion is shown for different bacteria strains. Evaluation of inclusion was performed using 18 different bacteria *(E. coli* n = 10), cultivated on non-selective TSA, CCA with CV supplement and CCA without supplement, as shown in Table 4. 

All 18 target bacteria showed pink and blue/purple growth on both CCA media and a similar amount of CFU in the non-selective TSA. Evaluation of exclusion with twelve different bacteria showed that the alternative method has huge advantages over the reference method shown in Table 5. Eight out of twelve bacterial strains containing *Pseudomonas aeruginosa*, *Aeromonas caviae*, *Aeromonas hydrophila*, *Acinetobacter iwoffii* and *Acinetobacter calcoaceticus* showed no growth on CCA with the Cefsulodin/Vancomycin supplement. *Acinetobacter baumannii* showed slightly reduced growth on supplemented CCA.

## 4. Conclusions

As it is very important in water analysis to get reliable results, the alternative method described in this study gives the opportunity to investigate water samples with high accompanying flora. During our exclusion study, *P. aeruginosa* and *Aeromonas* spp. growth was completely inhibited by the Cefsulodin/Vancomycin (CV) supplement. Morphological analysis showed a defined curvature of the CFUs of *E. coli*, which makes enumeration easier. Investigation of the false positive rate for this alternative method show values below 1%, which is a recommendable result. The only drawback of CV-supplemented CCA was a false negative rate of approximately 30%, which can lead to lower CFU counts during analysis. High values for specificity and sensitivity indicate that this new method is ready for application in water analysis. High values for specificity and sensitivity indicate that this method [17] is ready for application in water analysis in Austria

## Figures and Tables

**Table 1:** Settings of described analysis design.

Setting	Item	Factor 1:	Factor 2:	Factor 3:	Factor 4:
		Technician	Culture Media (Charge)	Incubation Temperature (°C)	Incubation Time (h)
1	1,2,3	1	a	34.5	21
2	1,2,3	2	b	37.0	24
3	4,5,6	1	b	34.5	24
4	4,5,6	2	a	37.0	21
5	7,8,9	1	b	37.0	21
6	7,8,9	2	a	34.5	24
7	10,11,12	1	a	37.0	24
8	10,11,12	2	b	34.5	21

**Table 2:** Measured values for pH, electrical conductivity and background microorganisms for each type of investigated water.

Category	Type	pH-Value	Electrical Conductivity (µS/m)	Colony Forming Units (CFU/mL)
	A	7.58	656	<1
α	B	6.84	382	<1
	C	6.98	227	>300
	D	7.72	443	<1
	E	7.27	834	<1
β	F	7.29	735	<1
	G	7.19	1088	<1
	H	8.04	4979	>300
	I	6.96	479	226
γ	J	5.31	503	<1
	K	5.25	495	<1
	L	6.33	4624	<1

**Table 3:** Performance characteristics for reference and alternative method.

		Reference Method	Alternative Method
Identification		n = 338	n = 251
Sensitivity [%]		85.3	82.2
Specificity [%]		97.3	98.6
False positive rate [%]		3.8	0.7
False negative rate [%]		10.7	31.4
Selectivity [%]		37.9	59.0
Repeatability			
	Category		
(*E. coli*)	α	0.055	0.075
	β	0.066	0.114
	γ	0.052	0.083
(*K. aerogenes*)	α	0.056	0.051
	β	0.060	0.065
	γ	0.057	0.078
Reproducibility			
	Category		
(*E. coli*)	α	0.055	0.075
	β	0.081	0.114
	γ	0.054	0.083
(*K. aerogenes*)	α	0.056	0.053
	β	0.060	0.065
	γ	0.060	0.080

**Table 4:** Inclusion study: reference method [A], alternative method [B] and non-selective agar TSA (tryptic soy agar) [C]. Reference method, alternative method and cultivation on TSA were performed in duplicates for each strain, with CFUs shown below.

Reference Strain	[A] (CFU)		[B] (CFU)		[C] (CFU)	
*Enterobacter cloacae* DSM 30054	64	67	59	56	67	69
*Escherichia coli* DSM 1103	85	83	68	75	85	80
*Escherichia coli* DSM 1576	86	82	88	85	85	79
*Escherichia coli* DSM 5210	74	81	71	80	78	85
*Escherichia coli* DSM 5695	61	55	53	56	52	56
*Escherichia coli* DSM 5923	48	51	45	47	53	49
*Escherichia coli* UTI DSM 10814	72	60	72	74	71	64
*Escherichia coli* DSM 11250	76	83	79	82	80	84
*Escherichia coli* DSM 18039	84	79	75	69	80	76
*Escherichia coli* DH5 alpha	79	84	78	75	85	87
*Escherichia coli* K12	48	51	46	49	52	60
*Klebsiella aerogenes* DSM30053	75	79	76	74	84	71
*Klebsiella* sp. DSM 4798	97	98	96	93	95	84
*Klebsiella pneumoniae* ATCC 4352	78	75	67	62	80	77
*Klebsiella pneumonae* DSM 789	58	55	59	53	64	68
*Klebsiella pneumonae* DSM 26371	67	76	65	72	69	59
*Klebsiella pneumonae* DSM 30104	62	74	65	58	58	63
*Klebsiella variicola* strain: ATCC 31488	78	86	84	85	76	81

**Table 5:** Exclusion study: reference method [A] and alternative Method [B]. CFUs of the reference strains were determined in duplicate for both methods.

Reference Strain	[A] (CFU)		[B] (CFU)	
*Aeromonas caviae* DSM 7323	74	89	0	0
*Aeromonas hydrophila* DSM 30187	85	79	0	0
*Acinetobacter baumannii* DSM 30007	93	86	52	61
*Acinetobacter calcoaceticus* DSM 30006	53	60	0	0
*Acinetobacter Iwoffii* DSM 2403	77	65	0	0
*Proteus mirabilis* DSM 788	70	77	65	63
*Pseudomonas aeruginosa* DSM 939	78	88	0	0
*Pseudomonas aeruginosa* DSM 1117	79	86	0	0
*Pseudomonas aeruginosa* DSM 1128	82	89	0	0
*Pseudomonas aeruginosa* DSM 50071	85	90	0	0
*Salmonella enterica* subsp. *enterica* DSM 17420	80	75	78	69
*Salmonella enterica* DSM 19587	66	61	54	49

## Data Availability

Not applicable.

## Supplementary Materials

The following supporting information can be downloaded at: https://www.mdpi.com/article/10.3390/microorganisms10122499/s1, Table S1: Water sample calculations; Table S2: Relative trueness data.
