# Correction: Schalli et al. Cefsulodin and Vancomycin: A Supplement for Chromogenic Coliform Agar for Detection of *Escherichia coli* and Coliform Bacteria from Different Water Sources. *Microorganisms* 2022, *10*, 2499

**DOI:** 10.3390/microorganisms13122859

**Published:** 2025-12-16

**Authors:** Michael Schalli, Sarah Maria Inwinkl, Sabine Platzer, Rita Baumert, Franz F. Reinthaler, Petra Ofner-Kopeinig, Doris Haas

**Affiliations:** 1 Department for Water-Hygiene and Micro-Ecology, D&R Institute of Hygiene, Microbiology and Environmental Medicine, Medical University of Graz, 8010 Graz, Austria; 2 Institute for Medical Informatics, Statistics and Documentation, Medical University of Graz, 8010 Graz, Austria; 3 Applied Hygiene and Aerobiology, D&R Institute of Hygiene, Microbiology and Environmental Medicine, Medical University of Graz, 8010 Graz, Austria; doris.haas@medunigraz.at

In the original publication [1], a correction has been made to the Abstract:

“For this reason, an alternative method with a Cefsulodin/Vancomycin (CV)-supplemented CCA should be evaluated regarding its application in water from different sources.”

In the first paragraph of the Section 1 Introduction, Orden SCO/778/2009 and Ribas et al. were not cited. The citations have now been inserted and should read as follows:

“In 2009, the Spanish government approved a method for the detection of *E. coli* and coliform bacteria based on an internal study, developed by Ribas et al. [17,18].”

The newly added references [17,18] are listed below. With this correction, the order of some references has been adjusted accordingly.

- 17. Orden SCO/778/2009, de 17 de Marzo, Sobre Métodos Alternativos Para el Análisis Microbiológico del Agua de Consumo Humano. Available online: https://www.boe.es/eli/es/o/2009/03/17/sco778 (accessed on 8 July 2025).
- 18. Ribas, F.; Tusell, E.; Vandevenne, C.A.; Niemela, S.I. Estudio para comprobar la equivalencia entre tres metodos alternativos y el método de referencia agar lactose TTC Tergitol 7 (ISO 9308-1:2000) en el recuento de bacterias coliforms y *Escherichia coli* en aqua. Informe dirigido al Ministerio de Sanidad y Consumo en apoyo del uso de métodos alternativos en el control microbiológico de las aguas de consume humano, de acuerdo con la Directiva Europea 98/83/CE, April 2006; pp. 1–60.

In the Section 4 Conclusions, reference [17] has been inserted, and the last sentence has been updated as follows:

“High values for specificity and sensitivity indicate that this method [17] is ready for application in water analysis in Austria.”

The authors state that the scientific conclusions are unaffected. This correction was approved by the Academic Editor. The original publication has also been updated.
